# An Update on Eradication of *Helicobacter Pylori* in Iran: A Review

**DOI:** 10.34172/mejdd.2024.389

**Published:** 2024-07-31

**Authors:** Mahboobe Ebrahimi, Sepehr Tirgar Fakheri, Faezeh Aeeni, Tarang Taghvaei, Mehdi Saberi Firoozi, Hafez Fakheri

**Affiliations:** ^1^Gut and Liver Research Center, Non-communicable Diseases Institute, Mazandaran University of Medical Sciences, Sari, Iran; ^2^General Physician, Private Clinic, Sari, Iran; ^3^Digestive Disease Research Institute, Tehran University of Medical Sciences, Tehran, Iran

**Keywords:** *Helicobacter pylori*, Eradication, Resistance, Iran, Concomitant therapy, Bismuth quadruple therapy

## Abstract

**Background:**

*Helicobacter pylori*, the most prevalent infection in the world, has great importance due to being related to peptic ulcer disease, gastric metaplasia, dysplasia, and even gastric adenocarcinoma or mucosa-associated lymphoid tissue (MALT) lymphoma. The standard *H. pylori* eradication regimen is based on antibiotic susceptibility testing. If susceptibility testing is not available, a standard treatment regimen will be recommended based on records of *H. pylori* resistance rates to antibiotics in a region or locally proven highly effective regimens (equal to or higher than 90% eradication rate). The aim of this review was to define suitable recommendations for local treatment in different cities of Iran.

**Methods:**

This review article consists of randomized controlled trials related to *H. pylori* eradication in Iran. Data including the kind of therapy, number of patients and per-protocol *H. pylori* eradication rates were recorded in data gathering forms. Data search was conducted in PubMed and Google Scholar databases from 2018 to December 2023.

**Results:**

According to our review of Iranian articles regarding first-line *H. pylori* eradication regimens, these treatment protocols could be recommended: Bismuth-clarithromycin quadruple therapy in Ardabil, bismuth-clarithromycin quadruple therapy with probiotics in Birjand, standard triple therapy in Ilam, bismuth quadruple therapy or bismuth triple therapy or concomitant regimen in Sari, sequential therapy in Tehran and bismuth quadruple therapy in Yazd. These regimes can be extended to other regions that have a similar situation. According to the reports of Iranian researchers, a quinolone-containing regimen (levofloxacin preferred) is recommended for second-line eradication therapy.

**Conclusion:**

Various *H. pylori* eradication regimens can be used as first-line therapy; however, choices for second-line therapy are limited. We recommend the quinolone-containing regimens as the preferred second-line therapy.

## Introduction

 Approximately more than 50% of the world’s population is infected by *Helicobacter pylori *organism.^1^ The prevalence of infection is higher in adults than in children.^2^ Over the past three decades, the number of infected people worldwide has declined due to improved health care and eradication treatments.^3,4^ The prevalence of *H. Pylori* infection is reported to be 70.4% in Ardabil,^5^ 54.4% in Ilam,^6^ 72.8% in Neyshabur,^7^ 87% in south of Tehran,^8^ 44.5% in Sari,^9^ 31.5% in Fardis-Alborz province,^10^ 85.3% in Mashhad^11^ and 50.9% in Rafsanjan.^12^ The pooled prevalence of *H. pylori* infection among Iranian adults was estimated to be 62% (61%- 64%).^13^

*Helicobacter pylori* infection always causes chronic gastritis. In most infected patients, there are not any symptoms, but it is potentially dangerous due to being related to peptic ulcer disease, gastric atrophy, metaplasia, dysplasia and gastric adenocarcinoma or mucosa-associated lymphoid tissue (MALT) lymphoma.^13,14^ *H. pylori* infection is also associated with extra gastric complications, such as respiratory diseases,^15^ central nervous system diseases,^16^ cardiovascular diseases,^17^ metabolic diseases,^18^ autoimmune diseases^19^ and hematologic disorders (refractory iron deficiency anemia,^20^ immune thrombocytopenic purpura^21^ and vitamin B12 deficiency).^22^

 In some particular situations,* H. Pylori* eradication is recommended even in patients without any symptoms to prevent further complications such as gastric cancer or ulcers.^13,14,23^ Multiple risk factors (environmental and genetic factors) are associated with gastric cancer.^24^ A pooled analysis study of the interaction between *H. pylori* infection and other modifiable risk factors of gastric cancer proved strong interactions between cigarette smoking and dietary salt in non-cardia cancer cases and between alcohol and low socioeconomic status of cardia cancer cases.^25^

 The most important issue is to determine which antibiotic combination shows the least failure and side effects. This question does not have a certain answer due to the increased antibiotic resistance and treatment failure worldwide over the past four decades.^26^ Therefore, empirical treatments without considering antibiotic susceptibility testing are one of the biggest threats to global health, according to the World Health Organization and the Council of the European Union.^27^ Drug resistance in *H. pylori* is mainly caused by chromosomal mutations, physiological changes (such as dysregulation of drug uptake), biofilms, coccoid formation, and other factors.^28^ In the 1990s, triple therapy had a high eradication success rate,^29^ but since 2000, it has become ineffective due to increasing microbial resistance.^30^ Overall prevalence of clarithromycin-resistant *H. pylori* was 27.53% worldwide in 54 different countries.^31^ Based on the results of a systematic review and meta-analysis in Iran, the rate of increase in resistance to metronidazole, amoxicillin, clarithromycin, ciprofloxacin, furazolidone, and tetracycline in *H. pylori* isolates was determined during three time periods (1999-2010, 2011-2016, 2017-2019). In a total of 66 studies investigating 5936 *H. pylori* isolates, the resistance of *H*. *pylori* to antibiotics from 2017 to 2019 in Iran was as follows: Metronidazole 62% (95% CI 57-67), clarithromycin resistance 36% (95% CI 26-47), amoxicillin resistance 36% (95% CI 26-47), tetracycline resistance 18% (95% CI 6-34), ciprofloxacin resistance 36% (95% CI 26-47), levofloxacin resistance 18% (95% CI 9-30), erythromycin resistance 29% (95% CI 12-50), furazolidone resistance 34% (95% CI 20-50), and combination of clarithromycin and metronidazole resistance 16% (95% CI 1-23).^32^ Resistance is a new concern in the world; at least two antibiotic resistance of *H. pylori* have been reported as 43% in one study and 75% in a recent study in Iran.^33,34^

 The ideal *H. pylori* eradication strategy is personalized treatment based on antibiotic susceptibility testing. Two main methods are culture-based or molecular-based polymerase chain reaction (PCR) and next-generation sequencing (NGS) of DNA. The culture-based method is time-consuming, and, on the other hand, molecular methods are expensive and unavailable in all hospitals.^35^ The significance of the test of cure has increased recently, along with the availability of post-cure susceptibility testing, which allows the physician to determine whether a previously susceptible infection has become resistant.^36^ Understanding the cause of treatment failure provides the clinician with information on obtaining high cure rates.^37^ For adequate adherence, careful patient orientation is needed for medications and how to decide in case of adverse events.^38^ If susceptibility testing is unavailable, eradication therapy should be based on locally proven, highly effective regimens.^35^ In Iran, antibiotic susceptibility testing is unavailable in all cities, and we must be aware of the cure rates of different regimens in our region. Therefore, if a proven regimen with 90% or higher per-protocol eradication is introduced in some areas of Iran, this regimen will be the first choice of treatment in the same location. But if such a regimen is not available in that area, the second choice for the first line of treatment could be a regimen with more than 85% eradication rate. In 2018, we published a review article on recommended regimens in Iran.^39^ Given the rise of microbial resistance, any regimen that has worked in the past will not necessarily work in the future. Therefore, we decided to define the update of *H. pylori* eradication treatment in Iran by examining the results of different treatment regimens in different regions of Iran during the last 5 years.

###  Data Collection

 This review article consists of randomized controlled trials related to *H. pylori* eradication in Iran. We searched through the PubMed database and Google Scholar website for studies published in English between 2018 and December 2023. In our search papers detected by title: (‘*Helicobacter pylori’* or ‘*H. pylori’*), and (Iran), and (‘eradication’ or ‘therapy’ or ‘*H. pylori* resistance’). Two gastroenterologists selected the relevant studies after reviewing the abstracts. Duplicated papers and non-randomized Persian-language papers were excluded. Finally, randomized controlled Persian trials in the English language published between 2018 and December 2023 were included in our analysis. Because the number of second-line *H. pylori* eradication regimens was very small, non-randomized clinical trials evaluating second-line therapies were also included. The data, including the kind of therapy, the number of patients and per-protocol *H. pylori* eradication rates, were recorded in data-gathering forms.

 The first line treatment regimens evaluated in Iran were: dual therapy, clarithromycin-containing triple therapy, modified clarithromycin-containing regimens (sequential, hybrid, reverse hybrid, and concomitant), bismuth-clarithromycin quadruple therapy, bismuth-furazolidone containing quadruple therapy, bismuth quadruple therapy, quinolone containing regimens. Dual therapy consists of high-dose amoxicillin and proton pump inhibitors (PPIs). Clarithromycin-containing triple therapy consists of PPIs, amoxicillin, and clarithromycin. Sequential therapy is defined as PPIs for 10 to 14 days plus amoxicillin in the 5 to 7 initial days of therapy, followed by clarithromycin and metronidazole or tinidazole in the last 5 to 7 days of therapy. Hybrid therapy consists of a PPI and amoxicillin for 10 to 14 days, adding clarithromycin and metronidazole for the final 5 to 7 days of treatment. Reverse hybrid reverses the sequence of drug administration.^40^ Simultaneously concomitant is all antibiotics that are administered at the same time without Bismuth.^1^

 Patients who were diagnosed with ulcer disease or gastritis caused by *H pylori* and were treated with eradication regimens were included in the study. In all studies, urease breath test or stool antigen test have been used to confirm the eradication of *H. pylori* 4-8 weeks after the end of treatment. Additionally, in cases of gastric ulcers, repeated endoscopy and biopsy of gastric tissue have been employed.

 An acceptable eradication rate in the per-protocol analysis was defined as equal to or higher than 90%, as defined by Graham,^41^ however, according to the Toronto Consensus Report, achieving an eradication rate > 85% can also be considered appropriate.^42^

## Results

###  First-Line pylori Eradication Regimens (2018-2023)

####  Dual Therapy 

 Two studies evaluated the effectiveness of this 14-day regimen in Ahvaz and Sari in the last 2 years. The per-protocol eradication rates were 72.4% and 82.2%, respectively. Therefore dual therapy was not effective in both cities.^43,44^

####  Clarithromycin-Containing Triple Therapy

 This regimen is used only if locally proven effectiveness is ≥ 90% or clarithromycin resistance is below 15%, as Maastricht VI advocated.^1^ Three studies were evaluated: with 14 days duration in Ilam and with 10 days duration in Sari and Mashhad. The per-protocol eradication rates in Ilam, Sari, and Mashhad were 90.5%, 83%, and 78%, respectively.^45-47^ This regimen has been effective as first-line therapy only in the Ilam study with 14 days duration.

####  Modified Clarithromycin-Containing Regimens

 Ten studies evaluated different modified clarithromycin-containing therapy without bismuth subcitrate. Four studies with concomitant therapy were done in Sari with 10-, 12- and 14-day treatment duration and the results showed the effectivity of 12-, and 14-day duration only. Among the different models of this regimen, concomitant therapy based on patient compliance and eradication rate has been better than other models. Sequential therapy did not have an acceptable eradication rate in Khorramabad and Isfahan studies, but it was acceptable in Tehran and appropriate in Mashhad (Table 1).

**Table 1:** Modified clarithromycin-containing therapy in Iran

**First author, Year**	**City**	**Treatment duration (days)**	**Per protocol eradication rate%**	**Regimen**	**Number of patients**
Kazemi,^48^ 2023	Sari	14	92.7%	Concomitant	164
Toosi^49^, 2023	Sari	14 14	89% 85.8 %	Concomitant reversed hybrid	164 153
Yadollahi,^44^ 2022	Sari	14	88.6%	Concomitant	114
Hajiani^43^, 2021	Isfahan	10	64.15%	Sequential	53
Mokhtare^50^, 2020	Tehran	10	90%	Sequential *	92
Bari^51^, 2020	Sari	10 12	85.9% 92.6%	Concomitant Concomitant	109 109
Ravarian^47^, 2019	Mashhad	10	87.8%	Sequential	66
Moradniani^52^, 2018	Khorramabad	14	76%	Sequential	100

*Second generation PPI used (esomeprazole).

####  Bismuth-Clarithromycin Quadruple Therapy

 Fourteen studies evaluated the efficacy of bismuth-clarithromycin quadruple therapy in Rasht, Ahvaz, Birjand, Tehran, Ardabil, Isfahan, Qom, and Kashan. Two studies achieved an appropriate eradication rate ( > 85%) according to Toronto Consensus Report ^42^ in Birjand and Tehran, and four studies were acceptable ( ≥ 90% eradication rate) in Birjand, Ardabil, and Rasht. This regimen had acceptable eradication in Rasht in the 2019 study but lost its effectiveness in the 2023 study in the same city (Table 2).

**Table 2:** Bismuth-clarithromycin quadruple therapy in Iran

**First author, Year**	**City**	**Treatment duration (days)**	**Per protocol eradication rate%**	**Regimen**	**Number of patients**
Mohtasham^53^, 2023	Rasht	14 14	75% 80%	PABC PABC + L^a^	225
Alavinejad^54^, 2023	Ahvaz	14	70%	PABC	409
Hajiani^43^, 2023	Ahvaz	14	76.1%	PABC	110
Naghibzade55, 2022	Birjand	14	86.5% 92.3% 94.2%	PABC PABC + L^a^ PABC + S^b^	52 52 52
Nikkhah^56^, 2021	Tehran	14	89.4%	OABC	100
Pourfarzi^57^, 2021	Ardabil	14	96.54%	OABC	1333
Ebrahimi,^58^ 2021	Isfahan	10	79.2%	PATCB^c^	53
Sarkeshikian^59^, 2020	Qom	14	65.45% 78.18%	EABC EABC + A^d^	110
Razavizadeh^60^, 2020	Kashan	14	68.8%	OABC	80
Arj^61^, 2020	Kashan	14	69.6%	OABC	102
Mansour-Ghanaei^62^, 2019	Rasht	14	91.9%	PABC	172
Sebghatollahi^63^, 2018	Isfahan	14	81.1%	PABC	75
Hajiani^64^, 2018	Ahvaz	14	83.8%	PABC	78

^a^ *Lactobacillus ruteri*; ^b^ *Saccharomyces boulardii*; ^c^ Sequential regimen; ^d^ A: atorvastatin. B, O, A, C, P, E, T: Bismuth sub-citrate, Omeprazole, Amoxicillin, Clarithromycin, Pantoprazole, Esomeprazole, Tinidazole.

####  Bismuth Quadruple Therapy

 Four studies evaluated the efficacy of bismuth quadruple therapy; two of them had eradication rate of ≥ 90%. This regimen is the suitable choice for the first-line treatment in Sari and has been successful in Yazd (high dose of amoxicillin and metronidazole) and has been appropriate in the Tehran study. In a recent study of bismuth triple therapy (high dose amoxicillin 1gm every 8 hours) in Sari, the per-protocol eradication rate was 92.8%^65^ (Table 3).

**Table 3:** Bismuth quadruple therapy in Iran

**First author, Year**	**City**	**Treatment duration (days)**	**Per protocol eradication rate%**	**Regimen**	**Number of patients**
Kazemi^48^, 2023	Sari	14	95.58 %	PABT	164
Nikkhah^56^, 2021	Tehran	14	88.1%	OBTM	100
Salmanroghani^66^, 2019	Yazd	14 14	95.5% 83.8%	OABM* OBTM	114

P, A, B, T, O, M.: Pantoprazole, Amoxicillin, Bismuth, Tetracycline, Omeprazole, Metronidazole *: metronidazole 500 mg and amoxicillin 1000 mg three times a day.

####  Bismuth-Furazolidone Containing Quadruple Therapy

 Six studies investigated furazolidone-containing regimens in Ardabil, Rasht, Gorgan, and Tehran.^57,62,67,68^ Except for the Ardabil study, which had an 85.3% eradication rate, other studies found that the eradication rate was below the acceptable value.

###  Quinolone-Containing Regimen

####  Levofloxacin Triple Therapy

 Two studies evaluated levofloxacin triple therapy, it was acceptable in Ilam with 95.3% eradication rate (levofloxacin, esomeprazole, and tinidazole). The same regimen was not effective in Sari with 10 days duration.^45,46^

####  Levofloxacin and Bismuth-Containing Regimens

 Five studies in Ahvaz, Rasht, Kashan, and Isfahan evaluated the efficacy of levofloxacin and bismuth-containing regimens with different durations. Only in Kashan it is appropriate with 14-day treatment duration (89.7% eradication rate). This regimen was not suitable for the first-line therapy in Isfahan, Rasht, and Ahvaz (Table 4).

**Table 4:** Quinolone-containing regimens

**First author, Year**	**City**	**Treatment duration (days)**	**Per protocol eradication rate%**	**Regimen**	**Number of patients**
**Bismuth quadruple therapy with levofloxacin**					
Alavinejad^54^, 2023	Ahvaz	14	81	PTBL	379
Mansour-Ghanaei^69^, 2022	Rasht	7 10	47.6 62.4	PABL	110
Arj^61^, 2020	Kashan	14	89.7	OBAL	87
Sebghatollahi^63^, 2018	Isfahan	14	70.8	PABT*, L	75
**Modified levofloxacin-containing therapy**					
Razavizadeh^60^, 2020	Kashan	10	78.8	OA,T* L (sequential)	80
Mokhtare^50^, 2020	Tehran	10	93	EA,T* L (sequential)	94
Hajiani^64^, 2018	Ahvaz	10	78.2	PA,TL (hybrid)	78
Moradniani^52^, 2018	Khorramabad	14	87.6	OA,L (hybrid)	100

O, P, E, B, A, L, T, T*: Omeprazole, Pantoprazole, Esomeprazole, Bismuth sub-citrate, Amoxicillin, Levofloxacin, Tetracycline, Tinidazole.

####  Modified Quinolone-Containing Therapies

 Four studies evaluated modified quinolone-containing therapy as sequential or hybrid regimens. The sequential type of this regimen was acceptable in Tehran, with 93% eradication rate. The hybrid type of this regimen was effective as an alternative choice in the first line in Khorramabad, but the sequential type was not effective in Kashan, and the hybrid type was not effective in Ahvaz (Table 4).

###  Second-Line Therapy for H. pylori Eradication 

 In patients with first-line treatment failure, five studies evaluated regimens for second-line therapy. A levofloxacin-containing regimen in Sari and Ilam for 14 days reported an acceptable eradication rate, and the Gorgan study reported an eradication rate of more than 85%. Quadruple therapy with bismuth and furazolidone was not effective in Gorgan. Triple therapy with rifampicin was not effective in Ilam also (Table 5).

**Table 5:** Second-line therapy for *H. pylori* eradication in Iran

**Year**	**City**	**1 line**	**2 lines**	**Duration**	**Per protocol eradication (%)**	**Numbers of patients**
Abangah^70^, 2019	Ilam	Unknown	OAR OAL	14 14	72 90	50 50
Seyyedmajidi^71^, 2019	Gorgan	Standard triple therapy	OAL OABF	14 14	86 78.8	60 60
Fakheri^72^, 2018	Sari	Modified clarithromycin-containing regimen	PAL	14	91.8	61

O, A, L, R, B, F: Omeprazole, Amoxicillin, Levofloxacin, Rifampicin, Bismuth sub-citrate, Furazolidone.

## Discussion and Recommendation

 The Maastricht VI / Florence Consensus Reportrecommends prescribing the bismuth quadruple therapy regimen as the first line of treatment in a region where the resistance of *H. pylori* to clarithromycin is unknown or more than 15%.^1^ In case of treatment failure, a levofloxacin-containing regimen is recommended. This Consensus Report recommends prescribing concomitant therapy as a second choice if bismuth quadruple therapy is not available. In case of failure of concomitant therapy in this Consensus Report, a regimen containing levofloxacin or bismuth quadruple therapy is recommended. American College of Gastroenterology Clinical Guideline^73^ and Toronto Consensus^42^ also have a similar recommendation. The fifth Chinese National Consensus recommends bismuth quadruple therapy in the first line of *H. pylori* eradication.^74^ The AGA Clinical Practice Update has recommended that the regimen containing rifabutin or levofloxacin be prescribed if the first line of treatment fails.^75^ Based on the findings of Iranian researchers regarding the first line of *H. pylori* eradication in regions such as Yazd, Sari, and Tehran, the bismuth quadruple therapy regimen and concomitant regimen in Sari are in accordance with the guidelines of Europe, Canada, and USA.^1,42,73^ In the global guidelines, there is no recommendation for the use of sequential therapy or regimens containing bismuth and clarithromycin/furazolidone. In recent Iranian studies in areas such as Ardabil, Birjand, and Tehran,^55-57^ the regimen containing bismuth and clarithromycin has been found efficient. In areas such as Ardabil,^57^ the regimen containing bismuth and furazolidone yielded satisfactory results. Moreover, in regions such as Tehran and Mashhad,^47,50^ sequential therapy, and in Ilam,^45^ standard triple therapy have been efficient and effective.

 The regimen containing bismuth and clarithromycin was acceptable in Rasht, according to a study in 2019, but this regimen lost its effectiveness in the same city after 4 years despite adding probiotic (*Lactobacillus ruteri*, 100 mg). It means the development of resistance over time, and the fact that a regimen is effective at one time does not mean it can be used all the time.

 The guidelines in the United States, Europe, Canada, and China recommended levofloxacin-containing triple therapy as a rescue therapy.^1,42,73-75^ In a number of Iranian studies, levofloxacin has been used in the first line of eradication. Despite success in some cases, it is a reasonable second-line regimen in Iran, but this regimen is not recommended as an initial treatment due to the high level of quinolone resistance.

 However, the local experience is also very important when considering the global guidelines and recommendations in each country. The guidelines of societies should be considered if they are in line with local experiences and may be ignored if they are in contrast with valid local experiences. Based on these findings, our recommendations for the first-line treatment in different regions of Iran are summarized in (Table 6). Regarding the second-line treatment, the limited studies that have been conducted show that regimens containing levofloxacin for 14 days are effective and can be recommended.

**Table 6:** Therapeutic options for first line *H. pylori* eradication in Iran – 2023

**Region**	**First option (≥90 eradication rate)**	** Second option (>85% eradication rate)**
Ardabil	14 days Bismuth-clarithromycin quadruple therapy	14 days Bismuth-furazolidone quadruple therapy
Birjand	14 days Bismuth-clarithromycin quadruple therapy + probiotics	14 days Bismuth-clarithromycin quadruple therapy
Ilam	14 days Standard triple therapy	
Mashhad		10 days Sequential
Rasht	*	
Sari	-12 &14 days Concomitant Therapy -14 days Bismuth Quadruple Therapy -14 days Bismuth Triple therapy	
Tehran	10 days Sequential	-14 days Bismuth-clarithromycin quadruple therapy -14 days Bismuth Quadruple Therapy
Yazd	14 days high dose Bismuth Quadruple Therapy	

* Bismuth-clarithromycin quadruple regimen was already acceptable in Rasht, but it is ineffective in a recent study.^53^

 It should be considered that in addition to antimicrobial resistance as the most important factor influencing the eradication rate, other factors such as patient’s compliance, efficacy of acid-suppressive drugs, age, and duration of therapy should be considered in evaluating the different regimens, which were not possible in this study.^76^ These could interfere with eradication rates, as higher doses and longer duration of therapy could increase the success rates. Furthermore, our study was limited to English reports of Iranian studies.

## Conclusion

 The ideal *H. pylori* eradication strategy is personalized treatment based on antibiotic susceptibility testing. If susceptibility testing isn’t available, standard treatment based on locally proven, highly effective regimens is recommended. Based on the recent 5 years studies, the recommended regimens for each region have been determined (Table 6). We recommend that physicians who practice medicine report their *H. pylori* eradication results in a local or national data bank for periodic evaluation and appropriate decisions based on the best recommendations according to local data and international guidelines. In case of acceptable eradication, it is recommended to continue prescribing the desired regimen, and if that regimen is not effective, it is recommended to stop prescribing it and use alternative regimens.

## References

[1] Malfertheiner P, Megraud F, Rokkas T, Gisbert JP, Liou JM, Schulz C (2022). Management of Helicobacter pylori infection: the Maastricht VI/Florence consensus report. Gut.

[2] Liou JM, Malfertheiner P, Lee YC, Sheu BS, Sugano K, Cheng HC (2020). Screening and eradication of Helicobacter pylori for gastric cancer prevention: the Taipei global consensus. Gut.

[3] Kakiuchi T, Matsuo M, Endo H, Nakayama A, Sato K, Takamori A (2019). A Helicobacter pylori screening and treatment program to eliminate gastric cancer among junior high school students in Saga Prefecture: a preliminary report. J Gastroenterol.

[4] Kusano C, Gotoda T, Ishikawa H, Moriyama M (2017). The administrative project of Helicobacter pylori infection screening among junior high school students in an area of Japan with a high incidence of gastric cancer. Gastric Cancer.

[5] Zandian H, Zahirian Moghadam T, Pourfarzi F, Malekzadeh R, Rezaei S, Ghorbani S (2023). Gastric troubles in Iran: the role of social and economic factors in Helicobacter pylori infection. Health Promot Perspect.

[6] Hemati S, Rahmatian A, Talee G, Bastani E, Rahmatian A, Shams M (2021). Prevalence of Helicobacter pylori and Its seasonality in Ilam, Iran. Jordan J Biol Sci.

[7] Salehi M, Ghasemian A, Shokouhi Mostafavi SK, Najafi S, Rajabi Vardanjani H (2017). Sero-prevalence of Helicobacter pylori Infection in Neyshabur, Iran, During 2010-2015. Iran J Pathol.

[8] Torabi R, Tebyanian H, Heiat M, Choopani A (2022). Seroepidemiological prevalence of Helicobacter pylori in the south of Tehran, Iran. Novel Clin Med.

[9] Maleki I, Mohammadpour M, Zarrinpour N, Khabazi M, Mohammadpour RA (2019). Prevalence of Helicobacter pylori infection in Sari Northern Iran; a population-based study. Gastroenterol Hepatol Bed Bench.

[10] Gharavi MJ, Zarei J, Roshani Asl P, Yazdanyar Z, Sharif M, Rashidi N (2022). Determination of Helicobacter pylori infection prevalence by non-invasive methods. Avicenna J Clin Microbiol Infect.

[11] Memar B, Ghaour-Mobarhan M, Eslami Hasanabadi S, Faroughi F, Ahadi M (2022). Unexpected high seroprevalence of Helicobacter pylori infection in Mashhad, Iran. Razavi Int J Med.

[12] Sadjadi A, Akbarpour E, Alimohammadian M, Masoudi S, Ghanbari R, Rajabi Pour Z (2022). A population-based cross-sectional study on the prevalence and associated risk factors of Helicobacter pylori infection in Rafsanjan, a low gastric cancer area in southeast Iran. Middle East J Dig Dis.

[13] Moosazadeh M, Bagheri Lankarani K, Afshari M (2016). Meta-analysis of the prevalence of Helicobacter pylori infection among children and adults of Iran. Int J Prev Med.

[14] Malfertheiner P, Megraud F, O’Morain CA, Gisbert JP, Kuipers EJ, Axon AT (2017). Management of Helicobacter pylori infection-the Maastricht V/Florence consensus report. Gut.

[15] Durazzo M, Adriani A, Fagoonee S, Saracco GM, Pellicano R (2021). Helicobacter pylori and respiratory diseases: 2021 update. Microorganisms.

[16] Baj J, Forma A, Flieger W, Morawska I, Michalski A, Buszewicz G (2021). Helicobacter pylori infection and extragastric diseases-a focus on the central nervous system. Cells.

[17] Jamkhande PG, Gattani SG, Farhat SA (2016). Helicobacter pylori and cardiovascular complications: a mechanism-based review on role of Helicobacter pylori in cardiovascular diseases. Integr Med Res.

[18] Azami M, Baradaran HR, Dehghanbanadaki H, Kohnepoushi P, Saed L, Moradkhani A (2021). Association of Helicobacter pylori infection with the risk of metabolic syndrome and insulin resistance: an updated systematic review and meta-analysis. Diabetol Metab Syndr.

[19] Wang L, Cao ZM, Zhang LL, Dai XC, Liu ZJ, Zeng YX (2022). Helicobacter pylori and autoimmune diseases: involving multiple systems. Front Immunol.

[20] Hudak L, Jaraisy A, Haj S, Muhsen K (2017). An updated systematic review and meta-analysis on the association between Helicobacter pylori infection and iron deficiency anemia. Helicobacter.

[21] Basta MN (2020). Immune thrombocytopenia is different from thrombotic thrombocytopenic purpura. J Obstet Anaesth Crit Care.

[22] Siddiqua T, Alam MN, Ahmad SM, Haque R, Ahmed T, Allen L, et al. Helicobacter pylori infection and vitamin B12 deficiency during early pregnancy in an urban slum in Bangladesh (P24-035-19). Curr Dev Nutr 2019;3(Suppl 1):nzz044.P24-35-19. 10.1093/cdn/nzz044.P24-035-19.

[23] Li H, Yang T, Tang H, Tang X, Shen Y, Benghezal M (2019). Helicobacter pylori infection is an infectious disease and the empiric therapy paradigm should be changed. Precis Clin Med.

[24] Yusefi AR, Bagheri Lankarani K, Bastani P, Radinmanesh M, Kavosi Z (2018). Risk factors for gastric cancer: a systematic review. Asian Pac J Cancer Prev.

[25] Collatuzzo G, Pelucchi C, Negri E, López-Carrillo L, Tsugane S, Hidaka A (2021). Exploring the interactions between Helicobacter pylori (Hp) infection and other risk factors of gastric cancer: a pooled analysis in the Stomach cancer Pooling (StoP) Project. Int J Cancer.

[26] Megraud F, Bruyndonckx R, Coenen S, Wittkop L, Huang TD, Hoebeke M (2021). Helicobacter pylori resistance to antibiotics in Europe in 2018 and its relationship to antibiotic consumption in the community. Gut.

[27] Allerberger F, Lechner A, Wechsler-Fördös A, Gareis R (2008). Optimization of antibiotic use in hospitals--antimicrobial stewardship and the EU project ABS international. Chemotherapy.

[28] Tshibangu-Kabamba E, Yamaoka Y (2021). Helicobacter pylori infection and antibiotic resistance - from biology to clinical implications. Nat Rev Gastroenterol Hepatol.

[29] Ishibashi F, Suzuki S, Nagai M, Mochida K, Morishita T (2023). Optimizing Helicobacter pylori treatment: an updated review of empirical and susceptibility test-based treatments. Gut Liver.

[30] Janssen MJ, Van Oijen AH, Verbeek AL, Jansen JB, De Boer WA (2001). A systematic comparison of triple therapies for treatment of Helicobacter pylori infection with proton pump inhibitor/ranitidine bismuth citrate plus clarithromycin and either amoxicillin or a nitroimidazole. Aliment Pharmacol Ther.

[31] Sholeh M, Khoshnood S, Azimi T, Mohamadi J, Kaviar VH, Hashemian M (2023). The prevalence of clarithromycin-resistant Helicobacter pylori isolates: a systematic review and meta-analysis. PeerJ.

[32] Sholeh M, Maleki F, Krutova M, Bavari S, Golmoradi R, Sadeghifard N (2020). The increasing antimicrobial resistance of Helicobacter pylori in Iran: a systematic review and meta-analysis. Helicobacter.

[33] Shokrzadeh L, Alebouyeh M, Mirzaei T, Farzi N, Zali MR (2015). Prevalence of multiple drug-resistant Helicobacter pylori strains among patients with different gastric disorders in Iran. Microb Drug Resist.

[34] Farzi N, Yadegar A, Sadeghi A, Asadzadeh Aghdaei H, Marian Smith S, Raymond J (2019). High prevalence of antibiotic resistance in Iranian Helicobacter pylori isolates: importance of functional and mutational analysis of resistance genes and virulence genotyping. J Clin Med.

[35] Price A, Graham DY, Tan MC (2023). Controversies regarding management of Helicobacter pylori infections. Curr Opin Gastroenterol.

[36] Sachs G, Meyer-Rosberg K, Scott DR, Melchers K (1996). Acid, protons and Helicobacter pylori. Yale J Biol Med.

[37] Graham DY (2023). Implications of the paradigm shift in management of Helicobacter pylori infections. Therap Adv Gastroenterol.

[38] Graham DY, Lew GM, Malaty HM, Evans DG, Evans DJ Jr, Klein PD (1992). Factors influencing the eradication of Helicobacter pylori with triple therapy. Gastroenterology.

[39] Fakheri H, Saberi Firoozi M, Bari Z (2018). Eradication of Helicobacter Pylori in Iran: a review. Middle East J Dig Dis.

[40] Temido MJ, Mbanze D, Almeida N, Oliveiros B, Gravito-Soares E, Figueiredo P (2023). Is hybrid therapy more efficient in the eradication of Helicobacter pylori infection? A systematic review and meta-analysis. Ann Clin Microbiol Antimicrob.

[41] Graham DY (2009). Efficient identification and evaluation of effective Helicobacter pylori therapies. Clin Gastroenterol Hepatol.

[42] Fallone CA, Chiba N, van Zanten SV, Fischbach L, Gisbert JP, Hunt RH, et al. The Toronto consensus for the treatment of Helicobacter pylori infection in adults. Gastroenterology 2016;151(1):51-69.e14. 10.1053/j.gastro.2016.04.006. 27102658

[43] Hajiani E, Hashemi SJ, Parsi A, Seyedian SS, Ramim T, Sabbaghan A (2022). Eradication efficacy of high-dose amoxicillin and proton pump inhibitor compared with quadruple therapy contained bismuth in the treatment of Helicobacter pylori. J Family Med Prim Care.

[44] Yadollahi B, Valizadeh Toosi SM, Bari Z, Fakheri H, Maleki I, Taghvaei T (2022). Efficacy of 14-day concomitant quadruple therapy and 14-day high-dose dual therapy on H pylori eradication. Gastroenterol Hepatol Bed Bench.

[45] Shahbazi S, Vahdat Shariatpanahi Z (2018). Comparison between daily single-dose triple therapy and conventional triple therapy on patient compliance and Helicobacter pylori eradication: a randomized controlled trial. Indian J Gastroenterol.

[46] Tirgar Fakheri S, Sadough A, Fakheri H. Comparing clarithromycin- and levofloxacin-containing triple therapies for first line Helicobacter pylori eradication Mazandran province, Iran. J Mazandaran Univ Med Sci 2019;29(176):1-9. [Persian].

[47] Ravarian B, Esmaeilzadeh A, Saeedi S, Moghbeli M, Ganji A (2019). Comparison of ten-day sequential and standard triple therapy for Helicobacter pylori eradication. Acta Med Iran.

[48] Kazemi A, Rahimi Petrudi A, Maleki I, Fakheri H, Taghvaei T, Hosseini V (2023). Effects of 14-days bismuth- and tetracycline-containing quadruple therapy with concomitant regimen for the first line Helicobacter pylori eradication. Caspian J Intern Med.

[49] Valizadeh Toosi SM, Maleki I, Hosseini V, Shokri-Afra H (2023). Non-inferiority of reverse hybrid regimen versus standard concomitant regimen for H pylori eradication in a randomized-controlled trial. Caspian J Intern Med.

[50] Mokhtare M, Nikkhah M, Behnam B, Agah S, Bahardoust M, Masoodi M (2020). A comparative study of the effect of 10-day esomeprazole containing levofloxacin versus clarithromycin sequential regimens on the treatment of Iranian patients with Helicobacter pylori infection. Indian J Pharmacol.

[51] Bari Z, Fakheri H, Taghvaei T, Yaghoobi M (2020). A comparison between 10-day and 12-day concomitant regimens for Helicobacter pylori eradication: a randomized clinical trial. Middle East J Dig Dis.

[52] Moradniani M, Mirbeik-Sabzevari Z, Jaferian S, Shafiezadeh S, Ehsani Ardakani MJ, Mirzaee Roozbahany M (2018). Levofloxacin based vs clarithromycin based sequential therapy in Helicobacter pylori eradication; a randomized clinical trial. Gastroenterol Hepatol Bed Bench.

[53] Mohtasham M, Joukar F, Maroufizadeh S, Mojtahedi K, Asgharnezhad M, Mansour-Ghanaei F (2023). Lactobacillus ruteri compared with placebo as an adjuvant in quadruple therapy for Helicobacter pylori eradication: a randomized, double-blind, controlled trial. Arab J Gastroenterol.

[54] Alavinejad P, Nayebi M, Parsi A, Abdelsameea E, Ahmed MH, Hormati A (2023). Levofloxacin + tetracycline quadruple regimen for eradication of Helicobacter pylori: a multicenter multinational randomized controlled trial. Middle East J Dig Dis.

[55] Naghibzadeh N, Salmani F, Nomiri S, Tavakoli T (2022). Investigating the effect of quadruple therapy with Saccharomyces boulardii or Lactobacillus reuteri strain (DSMZ 17648) supplements on eradication of Helicobacter pylori and treatments adverse effects: a double-blind placebo-controlled randomized clinical trial. BMC Gastroenterol.

[56] NikKhah M, Sohrabi M, Aghapour S, Faraji A, Khoonsari MR, Abedi H (2020). Comparison of two common quadruple therapy protocols for eradication of Helicobacter pylori in Iran: an open label, randomized, non-inferiority, clinical trial. Govaresh.

[57] Pourfarzi F, Zahirian Moghadam T, Zandian H, Malekzadeh R, Yazdanbod A (2021). Cost-effectiveness analysis of two routine therapeutic methods for Helicobacter pylori eradication: a Persian cohort-based study. Gastroenterol Hepatol Bed Bench.

[58] Rafiei R, Ebrahimi A, Hosseini S, Aliyari S, Torabi Z, Rafiei F (2021). Sequential therapy for Helicobacter pylori eradication with or without bismuth subcitrate. EC Pharmacol Toxicol.

[59] Sarkeshikian SS, Ghadir MR, Alemi F, Jalali SM, Hormati A, Mohammadbeigi A (2020). Atorvastatin in combination with conventional antimicrobial treatment of Helicobacter pylori eradication: a randomized controlled clinical trial. J Gastroenterol Hepatol.

[60] Razavizadeh M, Arj A, Madani M, Gilassi H (2020). Comparing the efficacy of sequential and standard quadruple therapy for eradication of H pylori infection. Acta Medica (Hradec Kralove).

[61] Arj A, Mollaei M, Razavizadeh M, Moraveji A (2020). The comparison of levofloxacin- and clarithromycin-based bismuth quadruple therapy regimens in Helicobacter pylori eradication. J Res Pharm Pract.

[62] Mansour-Ghanaei F, Samadi A, Joukar F, Tirgar Fakheri H, Hassanipour S, Ashoobi MT (2019). Efficacy and tolerability of fourteen-day sequential quadruple regimen: pantoprazole, bismuth, amoxicillin, metronidazole and or furazolidone as first-line therapy for eradication of Helicobacter pylori: a randomized, double-blind clinical trial. EXCLI J.

[63] Sebghatollahi V, Soheilipour M, Khodadoostan M, Shavakhi A, Shavakhi A (2018). Levofloxacin-containing versus clarithromycin-containing therapy for Helicobacter pylori eradication: a prospective randomized controlled clinical trial. Adv Biomed Res.

[64] Hajiani E, Alavinejad P, Avandi N, Masjedizadeh AR, Shayesteh AA (2018). Comparison of levofloxacin-based, 10-day sequential therapy with 14-day quadruple therapy for Helicobacter pylori eradication: a randomized clinical trial. Middle East J Dig Dis.

[65] Tirgar Fakheri S, Shokri-Afra H, Graham DY, Bari Z, Fakheri H (2024). A pilot study evaluating high dose esomeprazole, bismuth subcitrate and amoxicillin for eradicating Helicobacter pylori infection in Iran. Helicobacter.

[66] Salmanroghani H, Mirvakili M, Baghbanian M, Salmanroghani R, Sanati G, Yazdian P (2018). Efficacy and tolerability of two quadruple regimens: bismuth, omeprazole, metronidazole with amoxicillin or tetracycline as first-line treatment for eradication of Helicobacter pylori in patients with duodenal ulcer: a randomized clinical trial. PLoS One.

[67] Seyedmajidi MR, Hosseini SA, Vafaeimanesh J (2021). Comparing the effect of two low-dose and high-dose four-drug regimens of furazolidone in eradicating Helicobacter pylori. Middle East J Dig Dis.

[68] Noorbakhsh N, Nikpour S, Salehi M (2022). The efficacy and safety of furazolidone-bismuth quadruple therapy for Helicobacter pylori eradication with or without probiotic supplementation. Gastroenterol Hepatol Bed Bench.

[69] Mansour-Ghanaei F, Masihipour B, Fathalipour M, Hassanipour S, Sokhanvar H, Mansour-Ghanaei A (2022). The efficacy, safety, and tolerability of levofloxacin quadruple therapy for Helicobacter pylori eradication: a randomized, double-blind clinical trial. Evid Based Complement Alternat Med.

[70] Abangah G, Raughani A, Asadollahi P, Asadollahi K (2019). Comparison between the two drug regimens of PPI + amoxicillin + rifampicin and PPI + amoxicillin + levofloxacin for the treatment of H pylori infections resistant to the first line drug regimen among patients referred to Ilam clinics. Gastroenterol Hepatol Bed Bench.

[71] Seyyedmajidi M, Abbasi L, Seyedmajidi S, Hosseini SA, Ahmadi A, Hajiebrahimi S (2019). Levofloxacin-containing triple therapy versus bismuth-based quadruple therapy as regimens for second line anti- Helicobacter pylori. Caspian J Intern Med.

[72] Fakheri H, Bari Z, Taghvaei T, Hosseini V, Maleki I, Valizadeh SM (2018). The efficacy of levofloxacin-based triple therapy for Helicobacter pylori eradication after failure with clarithromycin-containing regimens. Govaresh.

[73] Chey WD, Leontiadis GI, Howden CW, Moss SF (2017). ACG clinical guideline: treatment of Helicobacter pylori infection. Am J Gastroenterol.

[74] Liu WZ, Xie Y, Lu H, Cheng H, Zeng ZR, Zhou LY (2018). Fifth Chinese National Consensus Report on the management of Helicobacter pylori infection. Helicobacter.

[75] Shah SC, Iyer PG, Moss SF (2021). AGA clinical practice update on the management of refractory Helicobacter pylori infection: expert review. Gastroenterology.

[76] Yan TL, Gao JG, Wang JH, Chen D, Lu C, Xu CF (2020). Current status of Helicobacter pylori eradication and risk factors for eradication failure. World J Gastroenterol.

